# Erlangen Score as a tool to predict progression from mild cognitive impairment to dementia in Alzheimer’s disease

**DOI:** 10.1186/s13195-018-0456-x

**Published:** 2019-01-05

**Authors:** Inês Baldeiras, Isabel Santana, Maria João Leitão, Daniela Vieira, Diana Duro, Barbara Mroczko, Johannes Kornhuber, Piotr Lewczuk

**Affiliations:** 1 Neurology Department, Centro Hospitalar e Universitário de Coimbra, Coimbra, Portugal; 2 Center for Neuroscience and Cell Biology, University of Coimbra, Coimbra, Portugal; 3 Faculty of Medicine, University of Coimbra, Coimbra, Portugal; 4 Department of Neurodegeneration Diagnostics, Medical University of Białystok, Białystok, Poland; 5 Department of Psychiatry and Psychotherapy, Lab for Clinical Neurochemistry and Neurochemical Dementia Diagnostics, Universitätsklinikum Erlangen and Friedrich-Alexander Universität Erlangen-Nürnberg, Schwabachanlage 6, 91054 Erlangen, Germany

**Keywords:** Alzheimer’s disease, Mild cognitive impairment—progression, Cerebrospinal fluid, Biomarker

## Abstract

**Background:**

The previously described and validated Erlangen Score (ES) algorithm enables interpretation of the cerebrospinal fluid (CSF) biomarkers of Alzheimer’s disease (AD), ordering them on an ordinal scale: from neurochemically normal (ES = 0) through improbable AD (ES = 1), possible AD (ES = 2 or 3), to probable AD (ES = 4). Here we assess the accuracy of the ES in predicting hazards of progression from the mild cognitive impairment (MCI) stage of AD to the dementia stage of the disease (Alzheimer’s disease dementia (ADD)) in a novel, single-center cohort.

**Methods:**

Baseline CSF biomarkers (amyloid beta (Aβ) 1–42, Aβ42/40, Tau, and pTau181), interpreted according to the ES, were used to estimate time to progression from the MCI stage of AD to ADD, conditional on age, gender, APOE ε4 genotype, and Mini Mental State Examination score in 144 MCI subjects, using the Extended Cox Model; the subjects were followed-up until they developed dementia or until they had been cognitively stable for at least 2 years. In addition, ES distributions were studied in 168 ADD cases and 66 neurologic controls.

Further, we stratified MCI patients into those who progressed to ADD faster (within 3 years, *n* = 47) and those who progressed slower (*n* = 74).

**Results:**

The distributions of the ES categories across the four diagnostic groups (Controls, MCI-Stable, MCI-AD, and ADD) were highly significantly different (Kruskal–Wallis *χ*^2^(df = 3) = 151.4, *p* < 0.001), with significant contrasts between each pair (*p* < 0.005), except between the ADD and the MCI-AD groups (*p* = 1.0). MCI patients with ES = 2 or 3 had 6–8 times higher hazards to progress to ADD compared to patients with ES = 0 or 1 in the first 3 follow-up years, and then their hazards decreased to those of the group with ES = 0 or 1. Patients with ES = 4 had hazards 8–12 times higher compared to the ES = 0 or 1 group. Faster progressors with ES = 2 or 3 had, in comparison to slower progressors, significantly lower Aβ1–42, Aβ1–40, and Aβ42/40, but comparable Tau and pTau181. A highly significant difference of the ES distributions between these two groups was observed (*p* < 0.001).

**Conclusions:**

Our current results reconfirm and extend the conclusions of the previously published report that the Erlangen Score is a useful tool facilitating interpretation of a complex pattern of the CSF AD biomarkers.

**Electronic supplementary material:**

The online version of this article (10.1186/s13195-018-0456-x) contains supplementary material, which is available to authorized users.

## Background

Decreased concentration of amyloid beta (Aβ) 1–42 peptide, decreased Aβ42/40 ratio, and increased Tau and pTau181 concentrations in cerebrospinal fluid (CSF) form the biomarker profile in Alzheimer’s disease (AD) [1]. This pattern reflects the two pathophysiologic processes of the disease: amyloidosis and neurodegeneration. Although the CSF biomarkers demonstrate very high diagnostic accuracy, and are routinely used as an AD diagnostics tool in some countries, their further acceptance is hampered by problems with comparability of the results obtained in different centers or even in one center but with different analytical platforms. This issue has already been addressed, to some extent, by efforts to standardize procedures for sample collection, measurements protocols and assay calibrators, but global acceptance of these novel approaches will certainly take time [2–5]. Moreover, as the AD CSF biomarkers are progressively used in daily clinical practice, interpretation of the results needs expertise and caution and the question remains how to interpret the information given by the biomarkers, that is often heterogeneous, with not all biomarkers falling into clear-cut normal/abnormal categories.

In order to harmonize the clinical interpretation of the CSF biomarker profiles, the Erlangen Score (ES) interpretation algorithm was first proposed [6], followed by other approaches, including logistic regression models [7], classification scales based on the number of pathologic biomarkers, like the Paris–Lille–Montpellier (PLM) scale [8, 9], or a nominal-scale A/T/N system [10]. In contrast to other interpretation algorithms, the ES enables ordering of the CSF patterns into five ordinal classes (0–4) with increasing degree of alterations. Analysis of the two pathologies (amyloidosis and neurodegeneration), as is done in the ES algorithm, can be seen from the perspective of topologic analysis of a geometric object; the concept of the dimension of a geometric object (in our case, interpretation of two independent groups of AD biomarkers) is the number of independent parameters (one for amyloid pathology and one for neurodegeneration) needed to pick out a unique point inside the object. However, any point specified by two parameters (amyloidosis and neurodegeneration) can instead be specified by one; in our case, the total score. Further, the ES introduces, for the first time in the interpretation of CSF AD biomarkers, the concept of border zone results. In a previous study, the ES was shown to correctly classify nondemented/mild cognitive impairment (MCI) subjects at increased risk of developing dementia in two independent, large-scale, multicenter cohorts (German Competence Network Dementias and US-ADNI), irrespective of the fact that they used entirely different sample handling protocols, disparate laboratory analytical platforms, and uncorrelated center-specific reference ranges [11]. Continuing the validation of the ES algorithm, in the current study we tested whether the ES is capable of accurately predicting hazards of progression from the MCI stage of AD to the dementia stage of the disease (Alzheimer’s disease dementia (ADD)) in a novel, single-center cohort.

## Materials and methods

### Study population

The population studied here derives from the Coimbra cohort described elsewhere [12]. AD dementia patients (ADD, *n* = 168) and MCI patients (*n* = 144) were recruited at the Dementia Clinic, Neurology Department of Coimbra University Hospital, according to the baseline and follow-up protocol already published [12]. Patients were enrolled in a systematic way and had biannual clinical observation and annual neuropsychological and functional evaluations. All patients underwent a thorough biochemical, neurological, and imaging (CT or MRI and SPECT) evaluation. At baseline, a neurologist completed a medical history with the patient and the caregiver, and conducted a general physical, neurological, and psychiatric examination as well as a comprehensive diagnostic battery protocol, including: cognitive instruments such as the Mini Mental State Examination (MMSE) [13] Portuguese version [14], the Montreal Cognitive Assessment (MoCA) [15] Portuguese version [16], the Alzheimer Disease Assessment Scale–Cognitive (ADAS-Cog) [17, 18] Portuguese version [19], and a comprehensive neuropsychological battery with normative data for the Portuguese population (Lisbon Battery for Dementia Assessment (BLAD) [20]) exploring memory (Wechsler Memory Scale subtests) and other cognitive domains (including language, praxis, executive functions, and visuo-construtive tests); and standard staging scales which provide objective information about subject performance in various domains, including the Clinical Dementia Rating scale (CDR) [21] for global staging, the Disability Assessment for Dementia (DAD) [22, 23] for evaluation of functional status, and the Neuropsychiatric Inventory (NPI) [24, 25] to characterize the psychopathological profile, including the presence of depression. All of the available information (baseline cognitive test, staging scales, clinical laboratory, and imaging studies) was used to reach a consensus research diagnosis. A similar approach was used for follow-up evaluations annually. Neither CSF biomarkers nor PET were taken into account in the diagnoses.

MCI patients included in this study were of the amnestic type and the diagnosis was made in accordance with the criteria defined by Petersen et al. [26] and more recently the framework for MCI due to AD, proposed by the NIA–AA criteria [27]. Petersen’s criteria were operationalized as follows: a subjective complaint of memory decline (reported by the subject or an informant); an objective memory impairment (considered when scores on standard Wechsler memory tests were > 1.5 SDs below age/education-adjusted norms) with or without deficits in other cognitive domains; normal general cognition suggested by normal scores in the MMSE and MoCA using the Portuguese cutoff scores [14, 28]; largely normal daily life activities, evaluated with a functional scale (DAD); and absence of dementia, indicated by a CDR rating of 0.5. All patients were in a stable condition, without acute comorbidities. As exclusion criteria for enrolment, we considered a significant underlying medical or neurological illness revealed by laboratory tests or imaging; a relevant psychiatric disease, including major depression, suggested in the medical interview and confirmed by the GDS; and CT or MRI demonstration of significant vascular burden [29] (large cortico-subcortical infarct; extensive subcortical white matter lesions superior to 25%; unilateral or bilateral thalamic lacunes; lacunes in head of caudate nucleus; more than two lacunes).

MCI cases were followed-up with this comprehensive protocol until they developed dementia or until they had been cognitively stable for at least 2 years, and were further dichotomized into those who were cognitively stable and those who developed ADD. No MCI patients who developed types of dementia other than ADD were included in this study. Progression to ADD required fulfilling clinical diagnostic criteria for probable AD (see later) and was operationalized as follows: objective evidence, by cognitive testing, of decline to dementia using the MMSE, MoCA, and ADAS-Cog scores and qualitative evaluation (i.e., impairment of memory plus another domain); and changes in global CDR rating from 0.5 to 1 or more, confirming the cognitive profile of dementia and loss of autonomy.

Dementia was diagnosed according to the 2011 NIA–AA criteria [30]. These cases were classified as probable ADD according to clinical and neuroimaging features.

We also included 66 neurological controls. Most of these individuals suffered from acute or chronic headaches, and a lumbar puncture (LP) was performed as part of their routine diagnostic evaluation in order to exclude bleeding or inflammation; in some cases, this procedure was considered in the investigation of a peripheral polyneuropathy. In both cases, the CSF cytochemical evaluation was normal and a major CNS disease was excluded. In their brief cognitive evaluation, the controls showed no subjective cognitive complaints, were independent in their instrumental daily life activities, and most of them were still professionally active.

### Laboratory determinations

CSF samples were collected from the subjects as part of their routine clinical diagnosis investigation. Preanalytical and analytical procedures were done in accordance with previously proposed protocols [31]. Briefly, CSF samples were collected in sterile polypropylene tubes, immediately centrifuged at 1800 × *g* for 10 min at 4 °C, aliquoted into polypropylene tubes, and stored at –80 °C until analysis. CSF Aβ1–42, Aβ1–40, Tau, and pTau181 were measured in the laboratory in Coimbra, in duplicate, by commercially available sandwich ELISAs (Innotest; Innogenetics/Fujirebio, Ghent, Belgium), as previously described [12].

In our hands, the mean intra-assay coefficients of variation (CVs) of these methods were 4.2% for Aβ1–42, 3.8% for Aβ1–40, 4.5% for Tau, and 4.2% for pTau181, and inter-assay CVs were 8.1% for Aβ1–42, 13.2% for Aβ1–40, 7.0% for Tau, and 7.2% for pTau181. In addition, external quality control of the measurements was performed under the quality control scheme of the Alzheimer’s Association Quality Control Program for CSF Biomarkers [32].

The reference values used in our laboratory, and applied in this paper, are 580 pg/ml for Aβ1–42, 0.068 for Aβ42/Aβ40, 250 pg/ml for Tau, and 37 pg/ml for pTau181.

Blood samples were also collected from MCI and AD patients for apolipoprotein E (APOE) genotyping. DNA was isolated from whole EDTA-blood using a commercial kit (Roche Diagnostics GmbH, Manheim, Germany), as described by the manufacturer. The analysis of the two polymorphisms at codons 112 and 158 of the *APOE* gene (rs429358 and rs7412) was performed by PCR-RFLP assay, as previously described [33].

### Erlangen Score

The ES was calculated according to the algorithm described elsewhere, and with border zone results defined as a pathologic result within 10% of the reference value (i.e., 10% decrease in Aβ1–42 and/or Aβ42/40, or 10% increase Tau and/or pTau181) [6, 11]. Briefly, a CSF result with all biomarkers normal is scored 0 points, and reported as “no neurochemical evidence for AD”; a pattern with border zone alterations in one biomarkers group (either Aβ or Tau/pTau, but not both) results in a score of 1, and is reported as “neurochemically improbable AD”; a CSF result with evident alterations in either Aβ metabolism (decreased Aβ1–42 concentration or Aβ1–42/Aβ1–40 ratio) or tau metabolism (increased concentrations of Tau and/or pTau181), but not both, is scored 2 points; and the same score of 2 points is given in the case of border zone alterations in the CSF biomarkers of both groups. A result with evident alterations in one biomarkers' group (either Aβ or Tau) accompanied by border zone alterations in the other group is scored 3 points; these two cases (with the ES = 2 or 3) are reported as “neurochemically possible AD”. Finally, evident alterations in both Aβ and Tau groups result in 4 points, and are reported as “neurochemically probable AD”. Additional file 1: Table S3 presents the ES in a form of a set of the if/then “commands”, easily implementable into laboratory software.

### Statistical analyses

If not stated otherwise, results of the continuous variables are presented as medians and interquartile ranges. Wherever appropriate, 95% confidence intervals (CIs) are reported. Distributions of categorical variables are presented as numbers or percentages per group. Differences between groups in continuous variables were tested with a *t* test, and differences in distribution of categorical variables classified by an ordinal variable with a Kruskal–Wallis rank test adjusting for ties, followed by Dunn’s pairwise comparison with Bonferroni correction.

Survival analyses were performed and visualized first with unadjusted Kaplan–Meier (KM) estimators, accompanied by Nelson–Aalen (NA) cumulative hazard estimators. The differences across the ES categories were tested with a log-rank test, including testing trend, and with a Wilcoxon test. Next, the hazard ratios (HRs) of progression to dementia were modeled by Cox regression, with the explanatory variables as stated in the corresponding models (M0–M3). The proportionality assumption was checked by the Schoenfeld residuals test under the null hypothesis that the HRs are time constant, and by visual inspection of the KM and NA curves. Since the hazard proportionality assumption was violated, the Extended Cox Model (ECM) was preferred over the Cox proportional hazard (CPH) model, with the ES category “neurochemically possible AD” included in the models as a time-varying variable interacting with the Heaviside function splitting the follow-up time into “less or equal than 3 years” and “more than 3 years”.

Logistic regression was used to model the conditional probability of progression to ADD at 3-year and 5-year follow-up. Linear regression was used to model concentrations of the CSF biomarkers in MCI subjects who progressed to ADD within 3 years (fast progressors) compared to those who did not (slow progressors), adjusted for age, gender, and the MMSE score. To test whether biomarker patterns in fast and slow progressors differ by ES categories, interaction terms of ES categories with the Heaviside function, defining fast and slow progression, were included in these models. Linear marginal (population-averaged) predictors, adjusted for the covariates kept at their means, were then post-estimated from the models and are presented with their 95% CI. *p* < 0.05 was considered statistically significant. All analyses were done with Stata 14.2 (StataCorp, College Station, TX, USA).

## Results

### Demographics of groups and results of CSF biomarkers

The demographics of the groups and the results of the CSF biomarkers are presented in Table 1; the detailed statistical comparisons of the four groups are published elsewhere [12]. Briefly, MCI-Stable patients (i.e., those who did not progress to dementia during the study) were significantly younger, had borderline significantly higher MMSE scores, and had significantly higher CSF Aβ1–42 and Aβ42/40 as well as significantly lower CSF Tau and pTau181 compared to the MCI-AD patients (i.e., those who progressed to ADD). The MCI-AD group was significantly enriched in *APOE* ε4 carriers. There were no significant differences in the CSF Aβ1–40 concentrations and in the gender distribution between the two groups.

**Table 1:** Demographic data and results of cerebrospinal fluid biomarkers

	Control group (*n* = 66)	MCI-Stable group (*n* = 74)	MCI-AD group (*n* = 70)	ADD group (*n* = 168)	*p* value^a^
Age (years)	57.5 (51–68)	65 (59–73)	71 (68–76)	68.5 (62–75)	< 0.001
Female gender (%)	59	66	63	67	0.73
APOE ε4 carriers (%)	NA	28	60	46	< 0.001
MMSE	NA	28 (25–29)	25 (23–28)	18 (14–21)	0.049
Maximum follow-up (years)	NA	16.0	10.0	NA	NA
Aβ1–42 (pg/ml)	852.9 (637.7–1041.1)	780.0 (572.2–949.0)	459.6 (352.3–603.8)	388.6 (308.2–532.7)	< 0.001
Aβ1–40 (pg/ml)	8833 (6537–11,471)	10,659 (7672–13,443)	10,379 (8088–12,265)	8410 (6803–11,489)	0.53
Aβ42/40	0.100 (0.074–0.127)	0.079 (0.044–0.108)	0.046 (0.035–0.059)	0.046 (0.034–0.065)	< 0.001
Tau (pg/ml)	178.3 (141.7–221.6)	215.5 (139.6–335.5)	448.0 (302.9–638.8)	459.7 (289.3–702.8)	< 0.001
pTau181 (pg/ml)	29.6 (22.5–37.0)	33.9 (24.0–46.7)	59.0 (38.3–75.0)	55.2 (39.1–79.8)	< 0.001

Continuous variables presented as median (interquartile range); proportions presented as percentage in a given group

*Aβ* amyloid beta, *AD* Alzheimer’s disease, *ADD* Alzheimer’s disease dementia, *APOE* apolipoprotein *E*, *MCI* mild cognitive impairment, *MMSE* Mini Mental State Examination, *NA* not applicable

^a^Contrasting MCI-Stable and MCI-AD groups. Continuous variables tested with two-tailed *t* test; proportions tested with Kruskal–Wallis test

### Distribution of Erlangen Score categories across diagnostic groups

The distribution of the five ES categories (0–4) across the four diagnostic groups is presented in Additional file 1: Table S1. Due to expectedly low numbers of cases in the categories with 1 and 3 points, and in line with the operating procedure of reporting the ES to physicians in the daily routine, we combined the categories with 0 or 1 points as “neurochemically improbable AD”, and the categories with 2 or 3 points as “neurochemically possible AD”. The distribution of these three categories (neurochemically improbable AD, neurochemically possible AD, and neurochemically probable AD) across the four diagnostic groups (Controls, MCI-Stable, MCI-AD, and ADD) is presented in Table 2. In both categorization approaches (i.e., into five and into three ES categories), highly significant differences were observed in the ES distributions across the groups (Kruskal–Wallis *χ*^2^(df = 3) = 151.4, *p* < 0.001), confirmed by contrasts between each pair of the groups (*p* < 0.005 in five group-to-group comparisons), except between the ADD and the MCI-AD groups (*p* = 1.0). Within the subgroup of control patients who tested positive (ES ≥ 2; *n* = 29), one was then lost for follow-up, one is now classified as vascular dementia, and 27 remain without cognitive impairment. Within AD patients with ES ≤ 2 (*n* = 35), six dropped out, two changed their classification to non-AD pathology (one vascular dementia and one hippocampal sclerosis), and 27 remain classified as AD.

**Table 2:** Distribution of Erlangen Score neurochemical categories across the four diagnostic groups

Diagnostic category	Erlangen Score neurochemical category		
	Improbable AD	Possible AD	Probable AD
Control group (*n* = 66)^a^	37 (56.1%)	28 (42.4%)	1 (1.5%)
MCI-Stable group (*n* = 74)^a^	29 (39.2%)	25 (33.8%)	20 (27.0%)
MCI-AD group (*n* = 70)^b^	3 (4.3%)	16 (22.9%)	51 (72.8%)
ADD group (n = 168)^b^	9 (5.4%)	35 (20.8%)	124 (73.8%)

Presented as number (percentage of the total number) in a given diagnostic group

*AD* Alzheimer’s disease, *ADD* Alzheimer’s disease dementia, *MCI* mild cognitive impairment

^a^Distribution significantly different (*p* < 0.005) from distributions in all three other categories

^b^Distribution significantly different (*p* < 0.005) from distributions in Control and MCI-Stable group, but not in ADD group (*p* = 1.0)

### Hazard ratios of progression from MCI to AD dementia estimated by Extended Cox Models

**Fig. 1:**
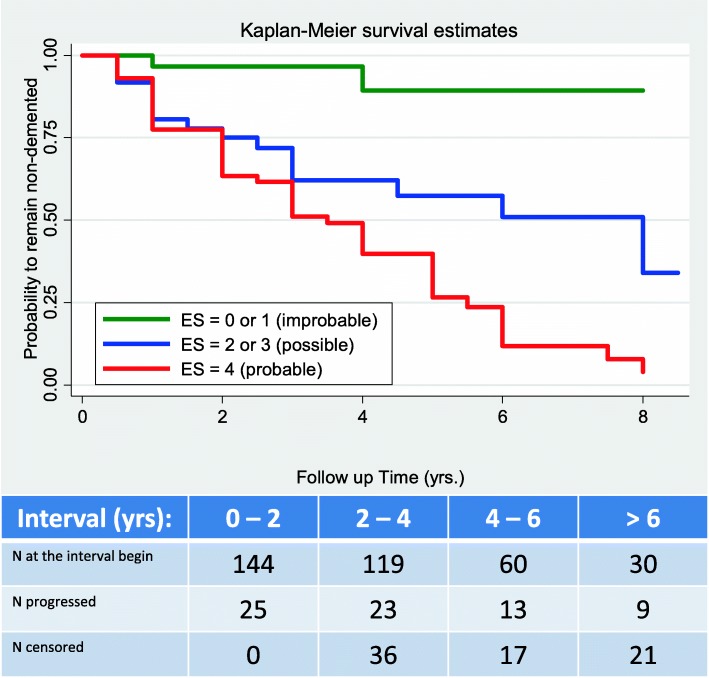
Kaplan–Meier survival curves of the three Erlangen Score (ES) categories. Note overlapping “possible” and “probable” curves in first 3 years, which then split apart with “possible” curve parallel to “improbable” curve. Table shows number of patients at beginning of each 2-year time interval, and numbers of patients having progressed to ADD or having been lost (censored) during each interval

Figure 1 presents the unadjusted KM survival curves in the three ES categories (neurochemically normal or improbable AD, neurochemically possible AD, and neurochemically probable AD). In addition, Additional file 1: Figure S1 presents the Nelson–Aalen estimators of the cumulative hazard functions of the three ES categories. Table 3 presents the HR estimates of the ECM, modeling the hazards of progression from MCI to ADD as the functions of the Erlangen Score (the first model, M0), plus the demographic covariates (age and gender, M1), supplemented further with the *APOE* genotype (M2), and finally completed with the cognitive status (MMSE score, M3). KM estimates turned out highly significantly different from one another (*p* < 0.001), with the value of the *χ*^2^ statistics from the log-rank test considerably larger than that from the Wilcoxon test (34.8 and 22.2, respectively). The latter finding speaks for smaller differences across the KM estimators at the earlier observation time and larger differences at the later observation time. This is consistent with overlapping of the neurochemically possible and neurochemically probable Nelson–Aalen cumulative hazard estimators in the first 3 years of observation, which then split apart leading to the possible AD NA curve parallel to the improbable AD NA curve, and considerably different from the probable AD NA curve.

**Table 3:** Extended Cox Models, estimating hazards of progression to AD dementia as functions of the covariates and with neurochemically possible AD category as a time-dependent variable, split into “before 3 years follow-up” and “after 3 years follow-up”

Covariate	M0 (*n* = 144)	M1 (n = 144)	M2 (*n* = 139)	M3 (*n* = 131)
Erlangen Score (reference category: Improbable AD)				
Possible AD				
Before 3-year follow-up	7.67 (2.08–28.27)*	7.75 (2.05–29.27)*	7.43 (1.95–28.37)*	5.68 (1.47–21.97)*
After 3-year follow-up	1.04 (0.20–5.47)	1.04 (0.20–5.50)	0.94 (0.18–4.99)	0.92 (0.17–4.91)
Probable AD	12.0 (3.67–39.46)*	12.3 (3.60–42.19)*	10.8 (3.09–37.38)*	8.09 (2.29–28.56)*
Age (years)	–	1.00 (0.97–1.03)	1.00 (0.97–1.03)	1.00 (0.96–1.03)
Female gender	–	1.10 (0.68–1.81)	1.07 (0.66–1.75)	1.05 (0.63–1.75)
APOE ε4 presence	–	–	1.42 (0.86–2.36)	1.48 (0.88–2.48)
MMSE	–	–	–	0.95 (0.87–1.02)
Log likelihood	− 284.0	− 283.9	− 280.6	− 264.5

Compare with the corresponding Kaplan–Meier curve (Fig. 1). Hazard ratios compared to reference category (neurochemically improbable, i.e. Erlangen Score = 0 or 1) presented together with the corresponding 95% confidence intervals

*AD* Alzheimer’s disease, *APOE* apolipoprotein *E*, *M0* model with ES as the only covariate, *M1* M0 supplemented with age and gender, *M2* M1 supplemented with APOE genotype, *M3* M2 supplemented with MMSE score, *MMSE* Mini Mental State Examination

*Statistical significance at *p* < 0.05 level, compared to the reference category

The trend across the three ES KM survival functions turned out highly significant (*χ*^2^(df = 1) = 33.11, *p* < 0.001).

Finally, the null hypothesis of the proportionality of the hazards was formally tested and rejected (*p* = 0.016). As a consequence, the ECM was preferred over the CPH model, with the estimated HR of the neurochemically possible AD category split into HR at the early (before 3 years) and at the late (after 3 years) follow-up time. Additional file 1: Table S2 presents the results of the CPH model, for sake of comparison with other studies.

Irrespective of all covariates (age, gender, *APOE* genotype, and MMSE score), patients with neurochemically possible AD (ES = 2 or 3) had about 6–8 times higher hazards to progress to ADD compared to the patients with neurochemically improbable AD (ES = 0 or 1) in the first 3 years, and then their hazards became comparable to those of the neurochemically improbable group. Patients with neurochemically probable AD (ES = 4) had hazards of progression to dementia 8–12 times higher compared to the neurochemically improbable AD group. Conditional on the covariates, the neurochemically probable AD group had hazards comparable to the neurochemically possible AD group in the first 3 follow-up years (M3: HR = 1.4, *p* = 0.31), which then became significantly higher (HR = 8.8, *p* < 0.005).

None of the other covariates tested in this study had significant HR estimates when adjusted for the Erlangen Score and/or other covariates. Of some relevance, perhaps, is that the MMSE score and the *APOE* genotype showed insignificant tendencies toward HR estimates different from 1, with each point increase of the MMSE score decreasing the hazard of progression by about 5–6% (M3: *p* = 0.16), and with the *APOE* ε4 genotype increasing the hazard of progression by about 50% (M3: *p* = 0.14), adjusted for other covariates.

### Probabilities of progression from MCI to AD dementia at 3 and 5 years after initial diagnoses

Table 4 presents two logistic regression models, estimating the probability to develop AD dementia within 3 and 5 years of the follow-up, respectively. Adjusted for age, gender, and the MMSE score, patients with neurochemically possible AD had about 9 and 4 times larger odds to become demented within 3 and 5 years, respectively, compared to the reference category, although these estimates are statistically weak (borderline significant (*p* = 0.048) after 3 years, and insignificant (*p* = 0.12) after 5 years). In contrast, patients with neurochemically probable AD had odds about 16 times larger at both follow-up time points, and these estimates were highly significant (*p* < 0.01 in both models). Adjusted for other covariates, the odds ratios of neither age nor gender nor MMSE score were significant.

**Table 4:** Logistic regression, modeling probability to progress to AD dementia within 3 and 5 years, respectively, as functions of age, female gender, and initial MMSE score

Covariate	Progression within 3 years (*n* = 116)	Progression within 5 years (*n* = 96)
Erlangen Score (reference category: Improbable AD)		
Possible AD	8.93 (1.02–78.04)*	3.90 (0.71–21.32)
Probable AD	16.32 (1.96–136.2)*	16.77 (2.95–95.40)*
Age (years)	1.00 (0.95–1.06)	1.01 (0.95–1.08)
Female gender	1.24 (0.52–2.92)	0.94 (0.34–2.59)
MMSE	0.90 (0.80–1.02)	0.91 (0.77–1.08)
Log likelihood	−66.6	−50.8

Odds ratios (95% confidence intervals) compared to the reference category (AD neurochemically improbable, i.e. Erlangen Score = 0 or 1)

*AD* Alzheimer’s disease, *MMSE* Mini Mental State Examination, n number of patients in a given model

*Statistical significance at *p* < 0.05 level, compared to the reference category

### CSF biomarker pattern in subjects progressing from MCI to ADD within shorter time (faster progressors)

Following the observation of the time-dependent hazard ratio to progress from MCI to ADD in the neurochemically possible AD category, we stratified MCI patients into a subgroup who progressed into ADD within 3 years (fast progressors, *n* = 47) and a subgroup of patients whose dementia-free follow-up time was longer than 3 years (slow progressors, *n* = 74). We observed a highly significant difference of the ES distributions between these two groups (*χ*^2^(df = 1) = 12.47, *p* < 0.001). In the neurochemically possible AD category, adjusted for age, gender, and MMSE score, faster progressors (*n* = 13) had, in comparison to slower progressors (*n* = 24), significantly lower CSF concentrations of Aβ1–42 [443 (95% CI 301–585) vs 830 (95% CI 726–932) pg/ml, *p* < 0.001] and Aβ1–40 [8338 (95% CI 6342–10,334) vs 11,347 (95% CI 9897–12,797) pg/ml, *p* = 0.018], and significantly lower Aβ42/40 ratio [0.054 (95% CI 0.040–0.069) vs 0.075 (95% CI 0.065–0.086), *p* = 0.023], but comparable concentrations of Tau [291 (95% CI 157–425) vs 275 (95% CI 177–372) pg/ml, *p* = 0.85] and pTau181 [39.1 (95% CI 25.9–52.2) vs 38.3 (95% CI 28.8–47.9) pg/ml, *p* = 0.93]. We did not observe differences in the CSF biomarkers or other variables between fast and slow progressors in either the neurochemically improbable or the neurochemically probable group. Adjusted for other variables, neither age nor gender nor MMSE score differed significantly between fast and slow progressors.

## Discussion

In this study, we confirmed our working hypothesis that the hazard of progression from the MCI stage to the dementia stage in AD strongly depends on the CSF biomarker pattern interpreted according to the Erlangen Score, and hence that the ES is a helpful tool as a predictor of dementia development in MCI subjects.

Expectedly, the distribution of the patients with different ES categories across the four diagnostic groups analyzed in this study showed statistically significantly higher proportions of patients with the highest ES outcome (ES = 4) in the ADD and the MCI-AD groups, compared to the neurologic controls and the stable MCI groups, which in turn showed a higher proportion of subjects with the lowest ES (0 or 1). The finding of essentially the same proportions of the ES categories in ADD and MCI-AD is fully in line with the currently most widely accepted model of the disease, which states that MCI-AD is a predementia stage in the continuity of ongoing AD pathology, and that the CSF biomarkers are capable of diagnosing the disease much before the development of the clinically observable dementia [34]. In our study, only 5% (12/238) of patients with AD were misclassified as “neurochemically improbable AD”, and only one of 66 neurologic controls was misclassified as “neurochemically probable AD”, which results in 95% sensitivity and 98.5% specificity. Intermediate distribution of the ES categories observed in the MCI-Stable group could be explained by the relatively short observation time; one cannot exclude that some of the MCI subjects so far stable will eventually have developed dementia in the future. Intermediate scores (ES = 2 or 3) observed in the neurologic controls as well as in MCI patients are attributable to imperfect accuracy of the AD biomarkers, rather than the weakness of the ES algorithm. Categorization of a patient within the “neurochemically possible AD” group should be seen, from that perspective, as a laboratory-driven recommendation to closely look at the results of other diagnostic modalities and to follow-up the patient to eventually disclose/confirm AD.

Empirical survival ES KM curves show highly significant differences, with a similarly highly significant trend. MCI patients classified as “neurochemically probable AD” had 8–12 times higher hazards to develop dementia than those classified as “neurochemically improbable AD”, adjusted for age, gender, MMSE score, and *APOE* genotype, and these hazard ratios were apparently time independent. On the other hand, the hazards associated with the demographic, cognitive, and genetic confounders were fully explained by the ES. Of particular relevance is that the hazards aligned with the two mostly accepted AD risk factors, age and *APOE* ε4 presence, are entirely explained by the ES categorization. Interestingly, in this study the hazard ratios in the “neurochemically possible” group turned out time dependent, showing significantly higher values in the first 3 years of follow-up, and then getting lower and comparable to the hazards in the “neurochemically improbable” group. This means that the probability to develop dementia in this group continuously increases but at a decreased rate after ca. 2–3 years. A large proportion (ca. 30%) of the MCI cases with “possible” scores developed dementia in the first 3 years of follow-up. This could be explained considering that: hazard ratios are relative metrics, normalizing the hazard in one category to the hazard in a reference category (“neurochemically improbable AD” in this case), the latter also showing some instable cases progressing to dementia (ca. 15% after the 4th follow-up year), even if their CSF results at the beginning of the study were normal; and the hazards, as they are considered in this study, are functions of the CSF results obtained once at the beginning of the observation and implicitly assumed to be constant over the whole follow-up time, which certainly does not need to be true. For example, it could happen that in a relatively short time after the LP (within 2–3 years), alterations in other biomarkers would append in addition to those already observed, changing a patient’s ES-based categorization from “possible” to “probable”. Interestingly, in this ES category, but not in the two others, significant differences of the CSF patterns between the fast and the slow progressors were observed, with amyloid biomarkers significantly lower in the former group. This observation stays in accordance with the hypothesis that an altered amyloid pathway triggers neurodegeneration, which then stimulates cognitive decline [35]; in such a scenario, biomarkers of neurodegeneration could have perhaps been observed in the CSF if the LP had been performed later in the course of the disease. In any case, it is plausible to conclude that the predictive value of the intermediate ES results (“neurochemically possible AD”) is most obvious within about 3 years following the CSF analysis, in contrast to clearly time-independent interpretation of either “improbable AD” or “probable AD”. Further, altered amyloid biomarkers, in this category, are particularly relevant as predictors of the MCI-ADD progression. This is also reflected by the results of the two logistic regression models, showing much higher odds ratios of having developed dementia after 3 years (8.9) compared to the odds ratios after 5 years (3.9) in the “possible” group, with consistently high odds ratios in the “probable” group (~ 16).

Compared to other classification and interpretation systems, the Erlangen Score shows clear advantages. It allows more precise stratification of patients into five categories with increasing degree of the CSF pathology, in contrast to the dichotomous approach (CSF normal/pathologic) applied by Hansson et al. [36]. Compared to regression-based approaches [7], the ES is much simpler; in everyday laboratory routine it does not need computer-based support at all—scoring of a CSF result consisting of four biomarkers takes less than 5 seconds for a moderately experienced person. Compared to the A/T/N classification [10], the ES stratifies subjects into classes on an ordinal scale, and not into purely nominal categories, which enables at least semi-quantitative correlation of the CSF findings with other metrics, like progression hazards, odds ratios, or survival-to-dementia time. Further, as an ordinal-scale classification system, the ES is able to take border-zone laboratory results into consideration, easily incorporating them into the interpreting algorithm. In contract, A/T/N is a purely nominal approach, which prevents existence of any “borderline” categories. Finally, compared to the PLM approach, which is based on the number of pathologic CSF biomarkers [9], the ES is more flexible, enabling inclusion of further potential biomarkers (as long as they reflect amyloid pathology or neurodegeneration at least on an ordinal scale) without necessity to redefine the ranges (i.e., the number of categories). Irrespective of the number of biomarkers considered, the ES will always classify the CSF patterns into five ordinal categories. As a matter of fact, in a previously published study the ES was successfully validated even when derived from three, instead of four, biomarkers available in the US-ADNI cohort, albeit with clearly less conclusive results compared to the validation based on four biomarkers available in the German CND cohort [11]. Flexibility of the ES extends its potential application beyond the CSF biomarkers; actually, results of every diagnostic modality, which analyzes amyloid pathology or neurodegeneration on a quantitative or at least semi-quantitative scale (like, for example, Aβ or Tau positron emission tomography), could be used to calculate the Erlangen Score. On the other hand, in comparison with the A/T/N classification, the ES is less informative, as the same score (in the categories 1, 2, and 3) can result from different combinations of biomarkers, and therefore have a different biological meaning. For instance, according to the ES, both patients with abnormal amyloid and normal neurodegeneration markers or with normal amyloid and abnormal neurodegeneration markers would score 2, and would therefore be given a similar interpretation in terms of CSF biomarker profile, whereas the A/T/N classification would attribute a completely different biological significance to these profiles. This could be of relevance when using biomarker profiles for patient recruitment in clinical trials, where specific pathological pathways are being targeted and therefore detailed information on which specific markers are altered is needed. To overcome this limitation, we postulate amending a numerical score with a graphic representation of the biomarkers’ pattern in the form of a table with the rows and the columns representing partial scorings for amyloid and neurodegezneration biomarkers, respectively, and with the total score in the table’s body (see Fig. 1 in [1]). Finally, it needs to be stressed, that as soon as laboratory (or method)-specific reference values for the biomarkers are established, the interpretational approach offered by the ES algorithm is independent of the center, laboratory platform, preanalytical sample handling procedures, and so forth. This characteristic might be seen as one of the most important advantages of the ES, since discrepancies in laboratory and method-specific cutoff values are one of the major problems hampering further acceptance of the CSF biomarkers as a routine AD diagnostic tool. This is clearly seen from the comparison of the interpretations in two completely independent cohorts, analyzed in two distinct laboratories, reported previously [11].

Our current results reconfirm the conclusions of the previously published report [11], even if the lower number of borderline results (particularly those with ES = 3) in the current study precludes a more detailed analysis of the relative hazards of this particular category. On the other hand, in contrast to the ADNI cohort, which has only three AD biomarkers available, in the current study we were able to evaluate the ES based on its original four-biomarker algorithm (i.e., including the Aβ42/40 ratio in addition to Aβ1–42, Tau, and pTau181). We believe that this is the reason for somehow higher hazard ratios in this study, particularly in the neurochemically probable AD category, which could be interpreted as a more clear separation of the categories achieved due to inclusion of the Aβ42/40 ratio, a biomarker well known to improve the accuracy of the AD diagnostics [12, 37–39]. On the other hand, in the current study the confidence intervals of the hazard ratios are broader, obviously as a consequence of the smaller number of the cases. Taken together, the same conclusions are obtained in both studies (US-ADNI and the current one) in spite of the fact that they have different settings (multicenter versus monocenter), they apply entirely different sample collection and handling protocols, they measure biomarkers with two different analytical methods (multiplexing and ELISA, respectively), and of course they use completely different sets of cutoff values.

This study is not without limitations, and perhaps the most serious is the low number of subjects with borderline biomarker concentrations, decreasing the sizes of the categories with ES = 1 or ES = 3. On the other hand, however, by definition the number of the borderline results should be low compared to the clear-cut results (ER = 0 or 2 or 4). Also, as seen in the tabulated part of Fig. 1, there is natural dropout of patients during the observation time, which limits the power of the conclusions that can be drawn beyond 5 years. Further, it is not the scope of this study to modify the current version of the Erlangen Score algorithm in such a way that it would apply different scoring weights to the biomarkers considered; for example, we are currently working on such a modification that would take into consideration that phosphorylated Tau seems to be a more specific AD biomarker than total Tau and that Aβ42/40 is obviously more accurate AD biomarker than Aβ1-42.

## Conclusions

Our results reconfirm and extend the conclusions of the previously published report that the Erlangen Score is a useful tool facilitating interpretation of a complex pattern of the CSF AD biomarkers; particularly, the Erlangen Score helps to understand CSF patterns in MCI patients progressing to AD dementia.

## Additional file

Additional file 1: **Table S1.** Distribution of Erlangen Score neurochemical categories (0–4) across the four diagnostic groups. **Table S2.** Hazard ratios in the Cox proportional hazard model. **Table S3.** Erlangen Score summarized in the form of a set of if/then commands, easily implementable into laboratory software. **Figure S1.** Nelson–Aalen cumulative hazard estimation. (PDF 322 kb)

## Abbreviations

AD: Alzheimer’s disease
ADAS-Cog: Alzheimer Disease Assessment Scale—Cognitive
ADD: Alzheimer’s disease dementia
APOE: Apolipoprotein
Aβ: Amyloid beta
CDR: Clinical Dementia Rating scale
CI: Confidence interval
CPH: Cox proportional hazard
CSF: Cerebrospinal fluid
CV: Coefficient of variation
df: Degree of freedom
DAD: Disability Assessment of Dementia
ECM: Extended Cox model
ELISA: Enzyme-linked immunosorbent assay
ES: Erlangen Score
HR: Hazard ratio
KM: Kaplan–Meier
LP: Lumbar puncture
MCI: Mild cognitive impairment
MMSE: Mini Mental State Examination
MoCA: Montreal Cognitive Assessment scale
NA: Nelson–Aalen
NIA-AA: National Institute on Ageing–Alzheimer’s Association
NPI: Neuropsychiatric Inventory
PCR-RFLP: Polymerase chain reaction–restriction fragment length polymorphism
PLM: Paris–Lille–Montpellier scale
SD: Standard deviation
US-ADNI: United States Alzheimer’s Disease Neuroimaging Initiative
