# Supplementary material for: Erlangen Score as a tool to predict progression from mild cognitive impairment to dementia in Alzheimer’s disease
Source: Alzheimers Res Ther. 2019 Jan 5;11:2. doi: 10.1186/s13195-018-0456-x (PMC6320577; doi:10.1186/s13195-018-0456-x)

Table S1. The distributions of the Erlangen Score neurochemical categories (0 - 4) across the four diagnostic groups. Presented are the numbers in a given diagnostic group.

	Erlangen Score					
Diagnosis	0	1	2	3	4	Total
AD	7	2	26	9	124	168
CTR	34	3	27	1	1	66
MCI-AD	3	0	13	3	51	70
MCI-St	26	3	20	5	20	74
Total	70	8	86	18	196	378

Table S2. Hazard ratios in the Cox Proportional Hazard model, i. e. under assumption of time-independent hazard ratios.

	HR	Std. Err.	z	P>|z|	[95% Conf.	Interval]
Erlangen Score:						
Possible	2.92	1.89	1.66	0.097	0.822	10.369
Probable	6.73	4.25	3.02	0.003	1.953	23.214
Age	1.00	0.02	0.18	0.860	0.969	1.038
Female gender	1.01	0.26	0.04	0.965	0.607	1.684
APOE e4 posit.	1.49	0.39	1.53	0.126	0.894	2.484
MMSE	0.94	0.04	-1.68	0.092	0.865	1.011

Table S3. Erlangen Score summarized in a form of a set of the if/then commands, easily implementable into laboratory software, based on the cut-off values established in the Laboratory in Coimbra, and presented in Materials and Methods, and "border zones" arbitrary defined as a pathologic result within 10% of the reference value.

Command line	Comment/Interpretation
If Aβ1-42 < 580 Or Aβ42/40 < 0.068 Then ES = ES + 1	Aβ1-42 or Aβ42/40 in border zone (i. e. slightly decreased, but not lower than reference value minus 10%).
If Aβ1-42 < 522 Or Aβ42/40 < 0.062 Then ES = ES + 1	Aβ1-42 or Aβ42/40 decreased below the border zone (i. e. lower than the reference value minus 10%).
If Tau > 250 Or pTau181 > 37 Then ES = ES + 1	Tau or pTau181 in border zone (i. e. slightly increased, but not higher than the reference value plus 10%).
If Tau > 275 Or pTau181 > 41 Then ES = ES + 1	Tau or pTau181 increased beyond the border zone (i. e. higher than 10% above the reference value).
If Tau > 1200 And pTau181 ≤ 41 Then ES = ES - 1	A case with a very high Tau, not accompanied by a corresponding increase in pTau181, decreases probability of AD-pathology in favor of the probability of underlying rapidly progressing neurodegeneration. Such cases were not observed in this particular study.

Figure S1. Nelson-Aalen cumulative hazard estimation. Note overlapping cumulative hazards in the "neurochemically possible" and "neurochemically probable" groups until ca. 3 year follow-up, which then clearly splits resulting in the cumulative hazard of the "possible" group still increasing, but at the rate comparable (parallel) to this in the "neurochemically improbable" (i. e. the reference) group.

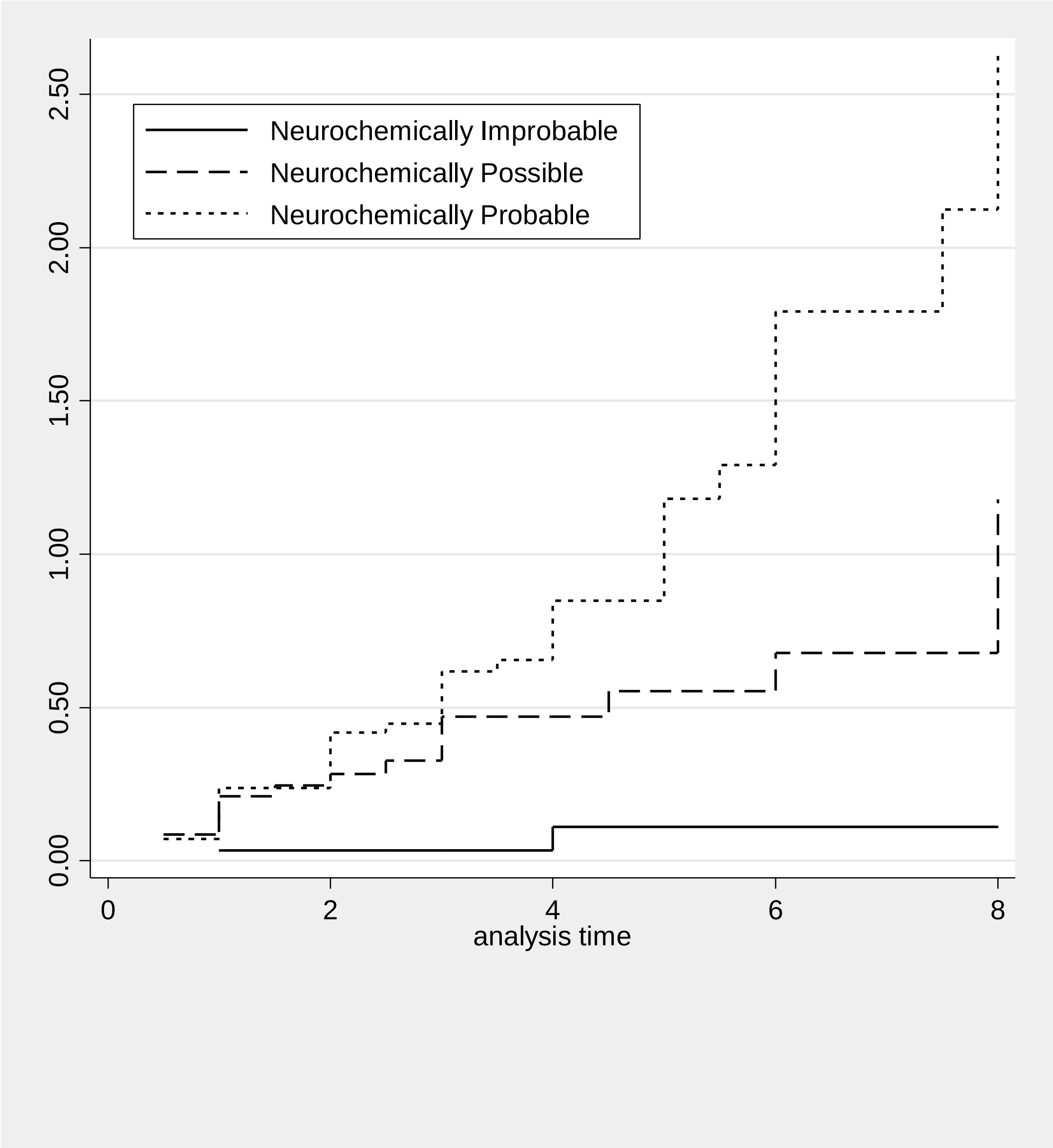

Supplement: Supplementary file 1 — Table S1. Distribution of Erlangen Score neurochemical categories (0–4) across the four diagnostic groups. Table S2. Hazard ratios in the Cox proportional hazard model. Table S3. Erlangen Score summarized in the form of a set of if/then commands, easily implementable into laboratory software. Figure S1. Nelson–Aalen cumulative hazard estimation. (PDF 322 kb) [file 13195_2018_456_MOESM1_ESM.pdf]
